# Isolated Renal Hydatid Cyst: A Rare Case

**DOI:** 10.7759/cureus.65143

**Published:** 2024-07-22

**Authors:** Manasa Suryadevara, Gaurav V Mishra, Pratapsingh Parihar, Mounika Suryadevara, Vadlamudi Nagendra, Rakshanda Agrawal, Anshul Sood, Saburi Singhania, Amol Rathod

**Affiliations:** 1 Radiodiagnosis, Datta Meghe Institute of Higher Education and Research, Wardha, IND; 2 Pulmonary Medicine, Ramaiah Medical College, Bengaluru, IND; 3 Interventional Radiology, Datta Meghe Institute of Higher Education and Research, Wardha, IND

**Keywords:** computed tomography (ct), abdominal pain, renal hydatid cyst, hydatid cyst, ultrasound (u/s)

## Abstract

Hydatid disease is a parasitic infection caused by a cestode from the Taeniidae family, by *Echinococcus multilocularis* or *Echinococcus granulosus*, predominantly occurring in the lungs and liver. Although the kidney can be involved in hydatid cysts, isolated kidney hydatidosis is very rare. Most cases present with non-specific complaints or remain asymptomatic for years. Hence, imaging is very useful in the diagnosis. Here, we report an isolated hydatid cyst involving the right kidney.

## Introduction

Hydatid disease, also referred to as echinococcosis, is a parasitic disease considered one of the important zoonotic infections with considerable public health importance, primarily due to the challenges associated with its diagnosis [1]. It is caused mainly by *Echinococcus granulosus* and, less frequently, by *Echinococcus multilocularis* [2]. As patients either remain asymptomatic or present with non-specific complaints, the diagnosis is very challenging. The adult *Echinococcus granulosus*, in its life cycle, resides in the small intestine of its definitive hosts, primarily dogs or, less frequently, other canids. The gravid proglottids release the eggs, which are excreted in the feces. When ingested by intermediate hosts, commonly sheep or other ruminants, these eggs reach the intermediate host’s small intestine, where the eggs release oncospheres that migrate through the lymphatic or portal circulation to the liver or, less frequently, other organs. In these sites, the embryos either die or develop into hydatid cysts. Humans can become infected when they consume water, vegetables, or other substances contaminated with *Echinococcus* eggs [3].

The clinical presentation of a cyst varies depending on its location, resulting in different symptoms from case to case. Some patients may remain asymptomatic for years. However, those with enlarging cysts are more likely to have symptoms. Additionally, unexpected signs and symptoms can be noticed if the cyst ruptures [4]. The liver serves as the primary line of defense and is the most commonly affected site, involved in about 75% of hydatid disease cases. Less commonly, hydatid cysts can affect the lungs, spleen, kidneys, brain, bones, and other rare anatomical sites [3]. Renal hydatidosis can occur when the *Echinococcus* larvae reach the kidney via direct invasion, the lymphatics, or the bloodstream. Kidney involvement typically indicates dissemination of the disease, as isolated renal hydatidosis is very rare [5]. Here, we report a case of isolated renal hydatid disease in a young man who presented with complaints of vague abdominal pain.

## Case presentation

**Figure 1:**
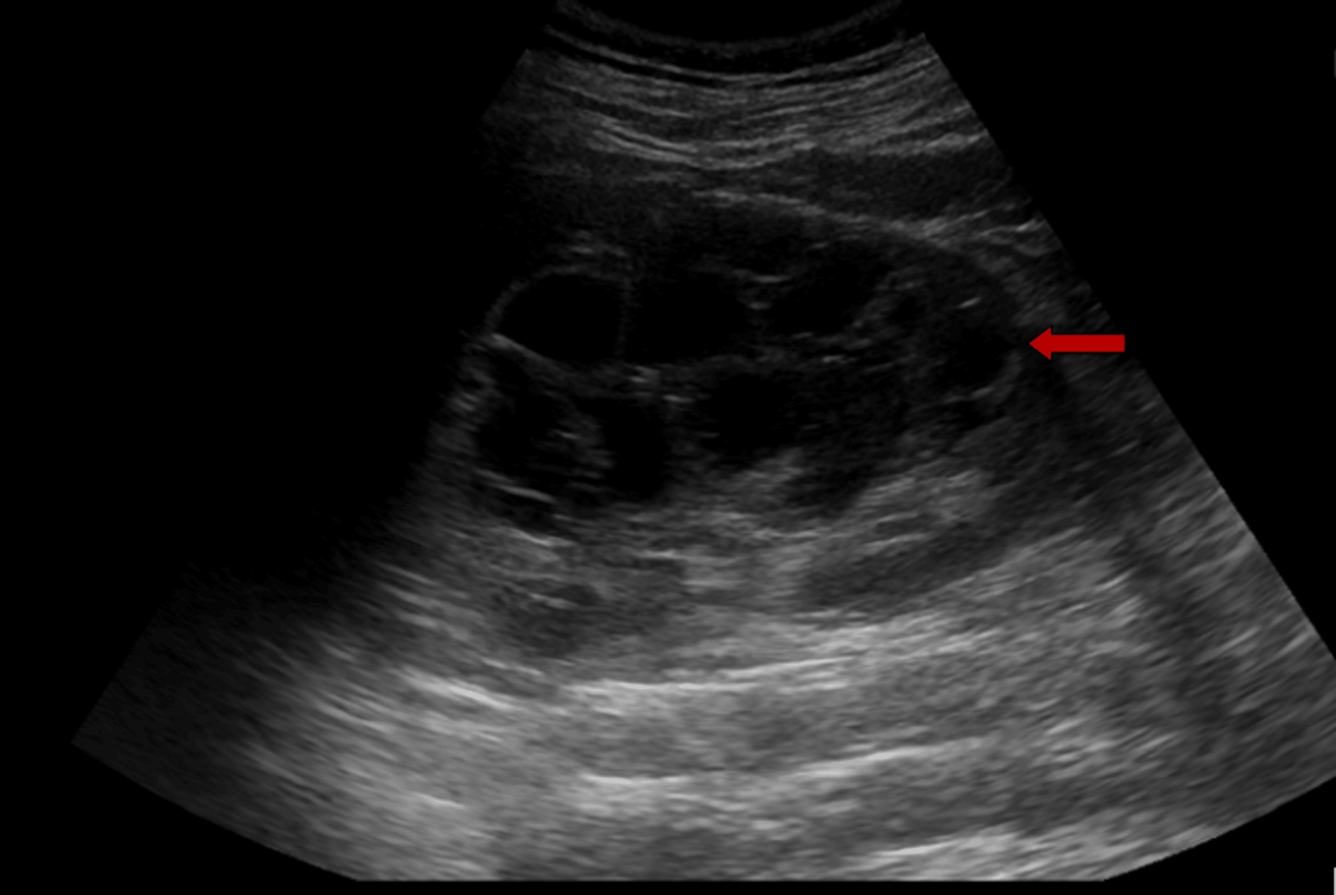
Ultrasound image showing multiloculated cystic lesion involving the upper, mid, and lower poles of the kidney (arrow).

A 31-year-old male with no significant surgical and medical history presented to our center with vague abdominal pain for 10 days. The pain was dull, aching, and non-radiating. The physical examination and hemogram revealed no positive findings. The urine analysis showed a high number of red blood cells and a little mucus. The patient underwent an ultrasound examination. The ultrasound scan revealed a large multilocular cystic lesion involving the upper, mid, and lower poles of the right kidney (Figure 1).

**Figure 2:**
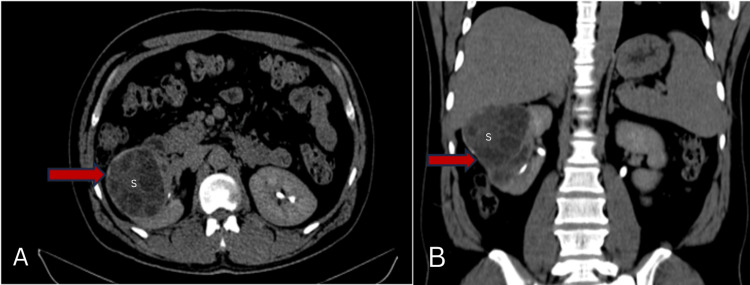
CT of the abdomen (A) axial and (B) coronal sections showing a multiseptated cystic lesion with an enhancing capsule involving the right kidney (arrow) showing sand within (S) extending up to the inferior surface of the liver.

**Figure 3:**
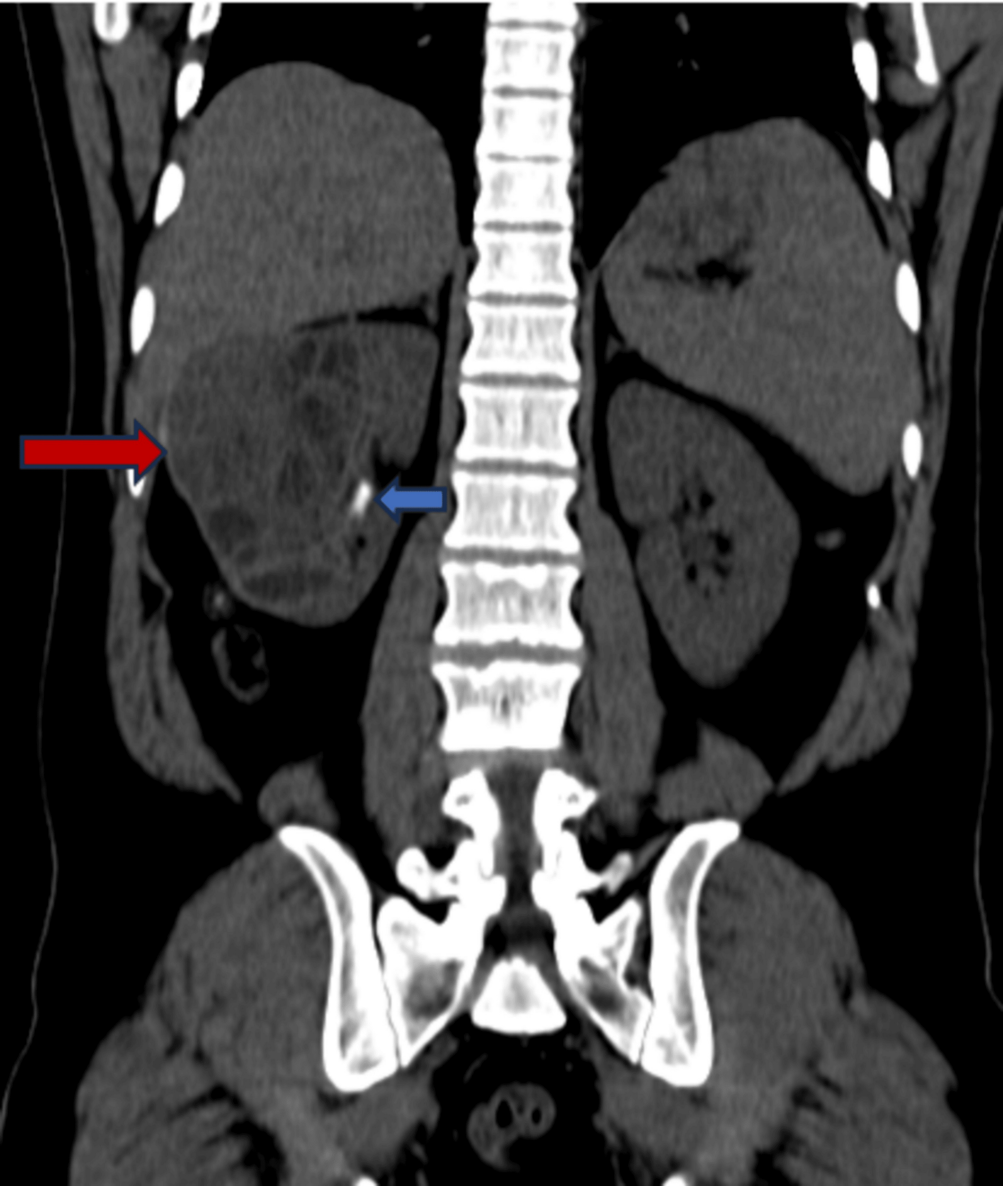
CT of the abdomen pre-contrast image showing a multiseptated cystic lesion involving the right kidney (red arrow) and a calculus (blue arrow) in the mid-pole of the right kidney.

The patient was advised CT which revealed a large multiseptated cystic lesion, a thick enhancing wall around with calcific foci within communicating with a calyx of the lower pole. The lesion was seen reaching up to the liver superiorly (Figure 2). The scan also revealed two calculi, one in the middle and the other in the inferior poles of the right kidney (Figure 3).

As the radiological evaluation gave the impression suggestive of a renal hydatid cyst and the patient gave a history of occasional contact with dogs till five years ago, the indirect hemagglutination (IHA) test was done, and the result was positive. The patient was advised of a surgical procedure which he refused. The patient was prescribed tablet albendazole 400 mg twice daily and was recommended follow-up.

## Discussion

Hydatid disease, also known as echinococcosis, is a parasitic infection caused by the *Echinococcus* species and can be transmitted to humans from animals. Canids are the definitive hosts, while humans are incidental hosts [2]. The symptoms can range from shivering and fever to symptoms related specifically to the involved organ. The symptoms may be silent initially, but complications may develop as the cyst grows [6]. Any system can be affected by the disease. Though the kidneys can be a part of the system involvement of hydatid disease, isolated renal hydatid disease is rare. The clinical presentation of kidney hydatid cysts can vary from being asymptomatic to causing loss of kidney function. The most common symptom patients present with is non-specific flank pain or vague abdominal pain, as seen in our patient. Additionally, hematuria or hydatiduria may occur if the cyst opens into the calyces. Hydatiduria is a rare presentation but is a pathognomonic sign of kidney hydatid cysts [2].

False-negative results can occur, although IHA is a sensitive test. The diagnosis can be confirmed by the positive serologic tests, but a negative test does not rule it out. Hydatid cysts can be visualized and assessed using ultrasonography, MRI, or CT. Sonography is the most commonly used modality, while CT or MRI may be beneficial when more detailed anatomical information is required. Ultrasonography typically reveals well-circumscribed uniloculated or multiloculated cystic lesions with prominent walls. The detection of daughter vesicles on ultrasonography or CT is also characteristic of hydatid cysts [2]. The treatment of hydatid cysts is multifaceted, including medicinal, percutaneous, or surgical approaches, with the most conservative therapy preferred. Studies indicate that cysts up to 5 cm can be treated solely with antiparasitic medication. For larger cysts, invasive treatment becomes necessary, and postoperative chemoprophylaxis is essential, typically with albendazole at 15 mg/kg daily for one to three months [7]. However, the treatment of choice for most cases is a renal-sparing procedure, as in our patient who refused the surgery [6]. According to the case reported by Moghtadaie et al. [6], medical therapy with the combination of praziquantel and albendazole can be used for patients who refuse the surgical procedure or cannot undergo surgery. In these cases, closer monitoring and follow-up are necessary due to the higher possibility of recurrence of cysts.

## Conclusions

Renal hydatid disease is a very rare entity. It is often detected very late, accounting for the fact that the patients either present with non-specific symptoms or even remain asymptomatic for a long duration. This can lead to loss of kidney function. Hence, it is necessary to consider the possibility of renal hydatid disease in patients presenting with non-specific pain in the flanks or urinary complaints such as hematuria, especially in endemic areas, even if the IHA test is negative. Mostly, the lesions are detected by ultrasound, after which additional imaging may be required for further evaluation. Timely therapy, monitoring, and follow-up are necessary to detect the recurrence of the cysts.
